# Medico-Legal Implications and Regulatory Frameworks of Regenerative Orthopaedics

**DOI:** 10.7759/cureus.42557

**Published:** 2023-07-27

**Authors:** Madhan Jeyaraman, Prince M Paul, Naveen Jeyaraman, Arulkumar Nallakumarasamy, Manish Khanna, Sankalp Yadav

**Affiliations:** 1 Orthopaedics, ACS Medical College and Hospital, Dr. MGR Educational and Research Institute, Chennai, IND; 2 Biotechnology, School of Engineering and Technology, Sharda University, Greater Noida, IND; 3 Regenerative Medicine, Indian Stem Cell Study Group (ISCSG) Association, Lucknow, IND; 4 Orthopaedics, South Texas Orthopaedic Research Institute (STORI Inc), Laredo, USA; 5 Forensic Medicine, Karuna Medical College, Palakkad, IND; 6 Orthopaedic Rheumatology, Dr. Ram Manohar Lohiya (RML) National Law University, Lucknow, IND; 7 Orthopaedics, Autonomous State Medical College, Ayodhya, IND; 8 Medicine, Shri Madan Lal Khurana Chest Clinic, New Delhi, IND

**Keywords:** orthopedics, lawsuits, regenerative orthopaedics, regulatory frameworks, medico-legal aspects

## Abstract

Regenerative orthopaedics has revolutionized traditional medicine, which represents a giant leap in science and research. The knowledge of the medico-legal implications and regulatory framework of this branch is vital for clinicians and researchers to go forward smoothly. This systematic review of the literature should shed light on these considerations and provide a comprehensive knowledge of the various implications and laws governing practice and research. The wide plethora of knowledge in the use of regenerative orthopaedics should be complemented by updated regulations and clinicians’ grasp of knowledge on regenerative medicine. The review focused on peer-reviewed published articles concerned with the topic and outlined common medico-legal issues and the current regulatory frameworks in various countries. The articles suggest that developed nations like the US have faced several lawsuits in this field, and a few countries in Europe like Italy and Germany, which were frontrunners in this field based on research, have fallen back due to emerging legal and regulatory policies. Undoubtedly, regenerative orthopaedics holds the key to future orthopaedics, but the world is skeptical of this concept, and laws and regulatory frameworks can curb it if not guided well. In India, this field has received prime attention, but at a slow pace when compared to the laws. After reviewing 113 articles, we analysed eight critically in this systematic review to emphasize the comparative global frameworks, daily medico-legal problems, and solutions for the branch of regenerative orthopaedics.

## Introduction and background

Regeneration in the field of medicine is always mind-boggling and creates hope for the future. When it comes to the musculoskeletal system, it is indeed more demanding and brings new horizons to the field of orthopaedics. Regenerative orthopaedics has emerged and evolved over the last few decades as a promising subspeciality [1]. With complicated orthopaedic conditions, illnesses and injuries that affect the musculoskeletal system are often painful and slow to heal, and traditional medications may not be effective for every patient. Regenerative orthopaedics utilizes healing substances that the body produces to trigger tissue regeneration [2,3]. Regenerative therapies are used to treat conditions such as arthritis, sports injuries, trauma, and degenerative spine diseases [4-9]. Degeneration to regeneration is always a welcoming aspect when it comes to the musculoskeletal system, thus opening up new horizons for a healthy society. It also encourages clinicians and researchers to update and bring out novel treatment procedures in regenerative orthopaedics. Time has evolved from the use of injected chondrocytes to treat chondral defects by Brittberg et al. in 1994 to the use of scaffolds, stem cells, cell-based therapies, and tissue engineering [10].

Regenerative orthopaedics is a challenging field that straddles medicine and surgery, and the treating clinician needs to be very aware of the legal scope of this field [11]. Doctors should be updated on the set of regulations and laws governing the practice for a safe and conflict-free profession. The medico-legal framework for procedures and novel drug delivery has to be well designed and executed in the emerging field of regenerative orthopaedics. This article would be unique in analysing the literature and data on the medico-legal implications, issues, and challenges of regenerative orthopaedics globally as well as in India.

## Review

Materials and methods

Research Question

What are the medico-legal implications and regulations in clinical practice and research of regenerative orthopaedics in India and other countries?

This study had two goals. First, we aimed to identify the medico-legal implications of regenerative orthopaedics globally as well as in India. Secondly, we aimed to design a few tips on preventing medico-legal issues and taking precautions to avoid legal hassles in the practice of regenerative orthopaedics.

Study Design

**Figure 1:**
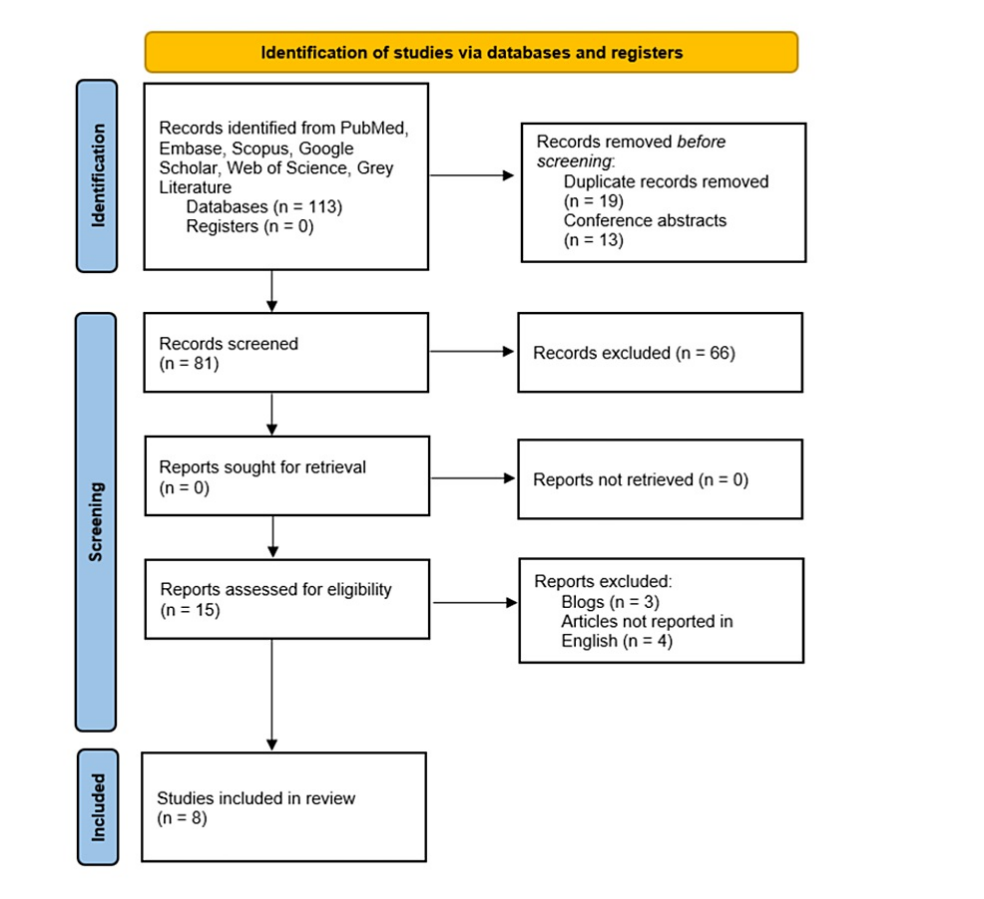
PRISMA flow diagram for the included studies PRISMA, Preferred Reporting Items for Systematic Review and Meta-Analysis.

A systematic review was conducted with the peer-reviewed journals published in indexed databases, conference proceedings, and authenticated literature on medico-legal aspects of regenerative orthopaedics until April 30th, 2022. The search for articles was done using PubMed, Embase, Scopus, Medline, Google Scholar, Web of Science, and grey literature. Through our search, a total of 113 articles were found related to the topics ‘medico-legal’, ‘regulations’, ‘regenerative orthopaedics’, ‘mesenchymal stromal cell’, and ‘mesenchymal stem cell’. This systematic review was conducted according to Preferred Reporting Items for Systematic Review and Meta-Analysis guidelines (PRISMA), as shown in Figure 1.

Inclusion Criteria

We included articles and literature from peer-reviewed, indexed journals, national and international conference proceedings, and authenticated laws and regulations related to the title topic until April 30th, 2022.

P - Published literature on medico-legal implications and regulatory frameworks for regenerative orthopaedics.

I - Regenerative therapies in orthopaedics.

C - No comparator group.

O - Medico-legal implications of regenerative orthopaedics.

Exclusion Criteria

We excluded blogs, opinion papers, press releases, social media videos, and theatrical reviews on this topic.

Analysis of Literature

A qualitative analysis of eight articles was done according to PRISMA guidelines.

Results

After thorough screening based on inclusion criteria and eligibility, eight articles were selected for review, as tabulated in Table 1.

**Table 1:** Details of eight articles that were selected for review

Author (year of publication)	Journal	Significance
Bhattacharyya et al. (2005) [12]	Journal of Bone and Joint Surgery American	Explains the need for informed consent in the surgeon’s chamber and must document the informed consent discussion within their dictated office or operative notes.
Keren-Paz and El Haj (2014) [13]	Tissue Engineering	Evaluates the relationship between the obligations to secure the patient's informed consent and to avoid clinical negligence for those who participate in innovative therapies.
Horner et al. (2018) [14]	NPJ Regenerative Medicine	Explores the value of a public health litigation strategy as a countermeasure against the exploitative practices of unproven stem cell injections by analysing stem cell lawsuits and comparing them with other major public health litigation efforts.
Katta (2018) [15]	JPMER	Raises concern about the physical, psychological, and financial harm to patients who received these cell-based therapies and the general lack of scientific evidence, professional accountability, and regulatory norms for these therapies.
Zamborsky et al. ​​​​​​​(2018) [16]	Ortopedia, Traumatologia, Rehabilitacja	Explains the indications for stem cell therapy in musculoskeletal disorders and the legal and ethical implications associated with such therapies.
Spinner et al. (2019) [17]	Techniques in Orthopaedics	Explains the challenges faced by product developers in terms of regulatory requirements and reimbursement processes before the product launch; explains the cost factor involved in such innovative cell therapies in orthopaedics.
Tingting et al. ​​​​​​​(2020) [18]	Health Policy	Emphasizes the various strategies to enhance approvals for regenerative medicine; explains the prevailing challenges and discrepancies in regulatory requirements among various nations.
Muthu et al. ​​​​​​​(2022) [19]	Bioengineering	Narrates Advanced Therapeutic Medicinal Product (ATMP) usage and regulatory frameworks both nationally and internationally.

Bhattacharyya et al., in their investigation of insurance companies, found out that the fundamental reason for lawsuits in the UK was improper consent before procedures [12]. It is important for the field of regenerative orthopaedics to formulate consent and to be taken in the right setting. Their results showed that documentation of appropriate informed consent in the office notes of the surgeon was associated with a decreased indemnity risk (P < 0.005). Keren-Paz et al. have mentioned the legal liability of clinicians for innovative practices and evaluated the relationship between the obligations to secure the patient’s informed consent and to avoid clinical negligence [13]. The researchers have pointed out that strict laws can lead to defensive medicine, which indirectly discourages innovative procedures like regenerative medicine (RM).

Horner et al. opined on the growth of unproven stem cell interventions in developed countries like the US and the need of the hour for policy changes and good regulatory frameworks to tackle this [14]. This is reflected in the lawsuits filed, which seek emergent attention and enforcement by authorities. Katta, in his comment on medico-legal issues in orthobiologics, has stated the freedom of doctors in choosing how to best treat their patients, but how professional norms restrain the scope [15]. Zamborsky et al. have underpinned the significance of informed consent, financial conflicts of interest, and the need for regulation of patents in stem cell treatment and enumerated various regulatory agencies in different parts of the world [16].

Spinner et al. have done an extensive search for market policies and regulatory methods for regenerative orthopaedics [17]. It is important to note that according to their study, the growing market and high cost of treatment can further lead to medical litigation, and the way out is policy and market regulation with standard authenticated practices. Tingting et al. have highlighted discrepancies in regulatory requirements and called for international coordination to standardize the terminologies, establish a universal regulatory pathway for RM to align evidence requirements to the highest standard, and allow international development for global access [18]. The regulatory agencies and the legal purview of different countries in RM are listed in Table 2. Muthu et al. have enlisted the regulatory frameworks in the US, Europe, and India for stem cell regulations. They have mentioned the flaws in laws and regulations and also the need to monitor the content of social media sites in advertising [19].

**Table 2:** Regenerative medicine's regulatory agencies and specific committees ATMP: Advanced Therapy Medicinal Products; IND: Investigational New drug; MA: Market Authorization; RM: Regenerative Medicine.

Country	Regulatory agency	Regenerative medicine (RM)-specific committees and their functions
United States [20]	Food and Drug Administration (FDA)	Centre for Biologics Evaluation and Research (CBER): Reviews all biologics for their risks and benefits, and analyses their use in the intended population after evaluation. Office of Tissue and Advanced Therapies (OTAT): Makes a mediation and platform for manufacturers and MA, conducts meetings for IND; Cellular, Tissue, and Gene Therapies Advisory Committee: Safety and effectiveness of RM are evaluated and reviewed. Tissue reference group (TRG): Issues and recommendations in applying RM for stakeholders
European Union [20]	The European Medicines Agency (EMA)	Committee for Advanced Therapies (CAT): Drafts opinions for ATMP MA before the Committee for Medicinal Products for Human Use (CHMP); certification of the quality of data for developing ATMP; Advice for classification, guidelines, training, and organization of scientific programs
Japan [20]	Pharmaceuticals and Medical Devices Agency (PMDA)	Cellular and Tissue-Based Products Subcommittee: Drugs and medical devices are evaluated and reviewed.
Canada [20]	Health Canada	Biologics and Genetic Therapies Directorate (BGTD): Authority for biological drugs, regulatory research with a focus on risk-benefit. Centre for Biologics Evaluation (CBE): biologics from tissues are regulated and evaluated. Centre for Evaluation of Radiopharmaceuticals and Biotherapeutics (CERB): gene therapy and technology-based therapy are regulated
Australia [20]	Therapeutic Goods Administration (TGA)	Advisory Committee on Biologicals (ACB): acts as an advisor to TGA regarding biological product use. The Office of Gene Technology Regulator (OGTR): Gene technology is controlled for its licensure, policy drafting, and regulation of law enforcement.
China [20]	National Medical Products Administration (NMPA)	Biological products clinical department under Centre for Drug Evaluation (CDE): MA for RM is reviewed, and clinical trials on biological products are evaluated.
India [20]	Central Drugs Standard Control Organization (CDSCO)	Department of Biotechnology (DBT): control on development, initiation of research, infrastructure, and regulatory frameworks. Cell Biology Based Therapeutic Drug Evaluation Committee (CBBTDEC): primarily scrutinizes and monitors new drugs.

Discussion

The field of medicine has been upgraded and updated since time immemorial. Every branch of medicine is looking for innovations, and the concept of RM is always promising. Now, the time has arrived when regeneration has grasped and taken over traditional practices. There are horizons of hope when the musculoskeletal system is regenerated. The real cutting edge of orthopaedics begins with the concepts of regeneration and orthobiologics, which in the current era have provided scope for practice and research. It is a giant leap for the branch of orthopaedics, to recognize this as a subspeciality, or rather a superspeciality.

Clinicians and researchers are geared up for this change. Regenerative orthopaedics has started to invade all ailments and disorders, including trauma [8]. But every innovation comes with hurdles. It is an Achilles heel for most practicing orthopaedic surgeons to try innovations if they are not authenticated or under the proper regulatory framework. Revitalizing musculoskeletal structures like bones and ligaments with less invasive procedures changes the standard of care. It is always important to note that ‘medical negligence’ exists only when standard and reasonable care does not exist. There is a considerable increase in musculoskeletal disorders and ailments due to the increase in the elderly population, which has led to disabilities and the use of steroids and anti-inflammatory drugs over the counter. Regenerative orthopaedics has changed this scenario with the application of stem cells and synthetic materials, leading to an interest in new studies [21].

The cost factor, effectiveness, and accessibility of these procedures for all needy populations are also matters of concern and debate. Medico-legal masquerades of regenerative orthopaedics hover around, with a quench to create trouble if meticulous and comprehensive guidelines are not followed. Pain reduction, speedy recovery, and improved outcomes stand out as good candidates for regenerative orthopaedics but proven and unproven therapies in wide use can tangle the way ahead.

The evolution of regenerative orthopaedics dates back centuries, from the use of prolotherapy to cell-augmented stimulation and regeneration injection therapies in the 21st century [2]. The future of regenerative orthopaedics is promising in providing relief, healing, and prevention of musculoskeletal diseases and disabilities. The aura of orthopaedics lies in regeneration, and it attracts not only patients but enthusiastic researchers to this field. There are obstacles and hindrances in this field when it comes to medico-legal considerations. The grey area of the regulatory framework and lacunae in the knowledge of medico-legal implications can lead to grave and diverse litigation. The clinicians and researchers should be knowledgeable, wise, and prudent enough to tackle these issues. Medico-legal implications and considerations that affect the clinicians in regenerative orthopaedics are highlighted below.

Patient selection: The ideal patient selection for appropriate treatment is crucial in this field. It should be based on scientific guidelines and indications. The procedures and interventions should be reliable and beneficial to the particular pathology.

The veracity of the treatment: The authenticity of the novel treatment should be checked and approved by the legal and regulatory framework of the place.

Complications: Any treatment procedure or intervention can have complications; only the extent and degree vary. The doctor should have the foresight of complications, disclosure, and accountability.

Competency: The competency of the treating doctor in such novel treatments is always questionable if adequate training is not done. The clinician must ensure up-to-date knowledge, practical training, and experience in this field, apart from relevant and valid qualifications.

Effectiveness: The efficacy and effectiveness of this form of treatment can be controversial if meticulous research and evidence do not come to the fore. The short-term and long-term benefits, along with the cost and risk of treatment, should be weighed before initiating and executing the treatment.

Consent: The requirement of informed written consent explaining all complications, benefits, alternatives, and risks of treatment to the patient by the treating doctor is the keystone to tying over most of the medico-legal issues. Proper counseling and communication should be done, which is the gold standard in any setting.

Documentation: The litigation and lawsuits in many countries against medical professionals were mainly because of faulty and incomplete documentation. Though the work is done duty-bound, a lack of documentation leads to a lack of evidence of commitment.

Liability: The liability for effective treatment falls on the clinician and the level of work done. In India, with the consumer protection act (CPA) clarification done by the Court of Law in April 2022, health care and its services are under the CPA's jurisdiction. It is to be noted that the more costly the treatment procedure, the more liable the clinician or health providers.

Medical negligence: The number of civil or criminal lawsuits against negligence is few globally, except in the US and other developed countries against regenerative orthopaedics. In India, statistically, there are no litigations or lawsuits filed against it, but rampant and advanced use of this booming field can invite litigations in the future. Further, in India, Central Drugs Standard Control Organization (CDSCO) and Drugs Controller General of India (DCGI) frameworks are in place, with research guidelines stated by the Indian Council of Medical Research (ICMR) [22-26].

## Conclusions

Orthopaedics has been transformed over decades, moving from conservative to surgical, replacement to preservation, radical to minimally invasive, and now to the role of regeneration. The torchbearers of regenerative orthopaedics should have a close look at and have a wide knowledge of the medico-legal issues and legal regulatory frameworks of their respective countries. In India, CDSCO and DCGI frameworks are in place, with research guidelines stated by the ICMR. There are lacunae and grey areas in medico-legal rules that always remain and are updated with the pace of growing science. The periodic re-evaluation and renewal of health policies and regulatory frameworks are key and should leave no stone unturned. The tightrope of medicine and law is a realm of the day, and any ignorance of the law is no longer an excuse after pitfall.

## References

[1] (2023). Biologic association aims to provide high-level evidence on regenerative medicine. https://www.healio.com/news/orthopedics/20200520/biologic-association-aims-to-provide-highlevel-evidence-on-regenerative-medicine.

[2] Vaish A, Murrell W, Vaishya R (2020). History of regenerative medicine in the field of orthopedics. J Arthrosc Surg Sport Med.

[3] Evans CH (2013). Advances in regenerative orthopedics. Mayo Clin Proc.

[4] Jeyaraman M, Muthu S, Jain R, Khanna M (2021). Autologous bone marrow derived mesenchymal stem cell therapy for osteonecrosis of femoral head: A systematic overview of overlapping meta-analyses. J Clin Orthop Trauma.

[5] Jeyaraman M, Muthu S, Jeyaraman N, Ranjan R, Jha SK, Mishra P (2022). Synovium derived mesenchymal stromal cells (Sy-MSCs): A promising therapeutic paradigm in the management of knee osteoarthritis. Indian J Orthop.

[6] Muthu S, Jeyaraman M, Ganie PA, Khanna M (2022). Is platelet-rich plasma effective in enhancing spinal fusion? Systematic overview of overlapping meta-analyses. Global Spine J.

[7] Jeyaraman M, Muthu S, Ganie PA (2021). Does the source of mesenchymal stem cell have an effect in the management of osteoarthritis of the knee? Meta-analysis of randomized controlled trials. Cartilage.

[8] Ranjan R, Kumar R, Jeyaraman M, Arora A, Kumar S, Nallakumarasamy A (2023). Autologous platelet-rich plasma in the delayed union of long bone fractures - A quasi experimental study. J Orthop.

[9] Jeyaraman M, Muthu S, Shehabaz S (2022). Current understanding of MSC-derived exosomes in the management of knee osteoarthritis. Exp Cell Res.

[10] Brittberg M, Lindahl A, Nilsson A, Ohlsson C, Isaksson O, Peterson L (1994). Treatment of deep cartilage defects in the knee with autologous chondrocyte transplantation. N Engl J Med.

[11] Jeyaraman M, Muthu S (2021). Inertia to integrate orthobiologics into orthopaedic practise: are we asking the right question?. Acta Sci Orthop.

[12] Bhattacharyya T, Yeon H, Harris MB (2005). The medical-legal aspects of informed consent in orthopaedic surgery. J Bone Joint Surg Am.

[13] Keren-Paz T, El Haj AJ (2014). Liability versus innovation: the legal case for regenerative medicine. Tissue Eng Part A.

[14] Horner C, Tenenbaum E, Sipp D, Master Z (2018). Can civil lawsuits stem the tide of direct-to-consumer marketing of unproven stem cell interventions. NPJ Regen Med.

[15] NA NA (2023). Proceedings of congress IBOSCON 2018. J Postgrad Med Educ Res.

[16] Zamborsky R, Kilian M, Csobonyeiova M, Danisovic L (2018). Regenerative medicine in orthopaedics and trauma: Challenges, regulation and ethical issues. Ortop Traumatol Rehabil.

[17] Spinner DS, Faulkner EC, Carroll MC, Ringo MC, Joines JW (2019). Regenerative medicine and cell therapy in orthopedics—health policy, regulatory and clinical development, and market access. Tech Orthop.

[18] Qiu T, Hanna E, Dabbous M, Borislav B, Toumi M (2020). Regenerative medicine regulatory policies: A systematic review and international comparison. Health Policy.

[19] Muthu S, Jeyaraman M, Kotner MB (2022). Evolution of mesenchymal stem cell therapy as an Advanced Therapeutic Medicinal Product (ATMP)-An Indian perspective. Bioengineering (Basel).

[20] Sawarkar S, Bapat A (2022). Global Regulatory Frameworks and Quality Standards for Stem Cells Therapy and Regenerative Medicines. Stem Cell Production: Processes, Practices and Regulations. https://books.google.co.in/books?id=A99mEAAAQBAJ&printsec=frontcover&source=gbs_ge_summary_r&cad=0#v=onepage&q&f=false.

[21] Ricci S, Ricci O, Tucci CE, Massoni F, Sarra MV, Ricci S (2012). [Regenerative medicine: orthopaedical applications and medico legal questions]. Clin Ter.

[22] (2023). India’s new drugs and clinical trials rules: An industry perspective. https://www.raps.org/News-and-Articles/News-Articles/2019/7/Indias-New-Drugs-and-Clinical-Trials-Rules-An-In.

[23] (2023). Guidelines | Indian Council of Medical Research | Government of India. https://main.icmr.nic.in/content/guidelines-0.

[24] Gogtay NJ, Ravi R, Thatte UM (2017). Regulatory requirements for clinical trials in India: What academicians need to know. Indian J Anaesth.

[25] (2023). Clinical research regulation for India | ClinRegs. https://clinregs.niaid.nih.gov/country/india.

[26] (2023). National guidelines for stem cell research- 2017 | Department of Biotechnology. https://dbtindia.gov.in/regulations-guidelines/guidelines/national-guidelines-stem-cell-research-%E2%80%93-2017.

